# Assessing the potential for improvement of primary care in 34 countries: a cross-sectional survey

**DOI:** 10.2471/BLT.14.140368

**Published:** 2015-01-28

**Authors:** Willemijn LA Schäfer, Wienke GW Boerma, Anna M Murante, Herman JM Sixma, François G Schellevis, Peter P Groenewegen

**Affiliations:** a NIVEL, Netherlands Institute for Health Services Research, PO Box 1568, 3500 BN Utrecht, Netherlands.; b Scuola Superiore Sant’Anna, Istituto di Management, Pisa, Italy.

## Abstract

**Objective:**

To investigate patients’ perceptions of improvement potential in primary care in 34 countries.

**Methods:**

We did a cross-sectional survey of 69 201 patients who had just visited general practitioners at primary-care facilities. Patients rated five features of person-focused primary care – accessibility/availability, continuity, comprehensiveness, patient involvement and doctor–patient communication. One tenth of the patients ranked the importance of each feature on a scale of one to four, and nine tenths of patients scored their experiences of care received. We calculated the potential for improvement by multiplying the proportion of negative patient experiences with the mean importance score in each country. Scores were divided into low, medium and high improvement potential. Pair-wise correlations were made between improvement scores and three dimensions of the structure of primary care – governance, economic conditions and workforce development.

**Findings:**

In 26 countries, one or more features of primary care had medium or high improvement potentials. Comprehensiveness of care had medium to high improvement potential in 23 of 34 countries. In all countries, doctor–patient communication had low improvement potential. An overall stronger structure of primary care was correlated with a lower potential for improvement of continuity and comprehensiveness of care. In countries with stronger primary care governance patients perceived less potential to improve the continuity of care. Countries with better economic conditions for primary care had less potential for improvement of all features of person-focused care.

**Conclusion:**

In countries with a stronger primary care structure, patients perceived that primary care had less potential for improvement.

## Résumé

**Objectif:**

Examiner la perception des patients quant au potentiel d'amélioration des soins de santé primaires dans 34 pays.

**Méthodes:**

Nous avons mené une enquête transversale sur 69 201 patients qui venaient juste de consulter des médecins généralistes dans des établissements de soins de santé primaires. Les patients ont évalué cinq caractéristiques des soins de santé primaires axés sur la personne: accessibilité/disponibilité, continuité, exhaustivité, implication du patient et communication entre le médecin et le patient. Un dixième des patients ont classé l'importance de chaque caractéristique sur une échelle allant d'un à quatre, et neuf dixièmes ont noté leur expérience des soins reçus. Nous avons calculé le potentiel d'amélioration en multipliant la proportion d'expériences négatives des patients avec le score moyen d'importance danschaque pays. Les scores ont été répartis en potentiels d'amélioration faible, moyen et élevé. Nous avons effectué des corrélations par paire entre les scores d'amélioration et les trois dimensions de la structure des soins de santé primaires: gouvernance, conditions économiques et constitution de la main-d'œuvre.

**Résultats:**

Dans 26 pays, une ou plusieurs caractéristiques des soins de santé primaires présentaient des potentiels d'amélioration moyen ou élevé. L'exhaustivité des soins avait un potentiel d'amélioration moyen à élevé dans 23 des 34 pays. Dans tous les pays, la communication entre le médecin et le patient présentait un potentiel d'amélioration faible. Une structure globale plus forte des soins de santé primaires était corrélée avec un potentiel plus faible d'amélioration pour la continuité et l'exhaustivité des soins. Dans les pays avec une gouvernance plus forte des soins de santé primaires, les patients percevaient un moindre potentiel pour améliorer la continuité des soins. Les pays présentant de meilleures conditions économiques pour les soins de santé primaires avaient un moindre potentiel pour l'amélioration de toutes les caractéristiques des soins de santé axés sur la personne.

**Conclusion:**

Dans les pays avec une structure plus forte des soins de santé primaires, les patients perçoivent un moindre potentiel d'amélioration pour les soins de santé primaires.

## Resumen

**Objetivo:**

Investigar las percepciones de los pacientes acerca de la mejora en la atención primaria en 34 países.

**Métodos:**

Se realizó una encuesta transversal de 69 201 pacientes que acababan de visitar médicos generales en centros de atención primaria. Los pacientes evaluaron cinco características de la atención primaria centrada en la persona: accesibilidad y disponibilidad, continuidad, exhaustividad, implicación del paciente, así como comunicación entre médico y paciente. Una décima parte de los pacientes clasificó la importancia de cada característica en una escala de uno a cuatro y nueve de cada diez pacientes evaluaron sus experiencias de la atención recibida. Se calculó el potencial de mejora multiplicando la proporción de experiencias negativas de pacientes con la puntuación media de la importancia en cada país. Las puntuaciones se dividieron en potencial de mejora bajo, medio y alto. Se realizaron correlaciones por pares entre las puntuaciones de mejora y las tres dimensiones de la estructura de atención primaria, a saber, gestión, condiciones económicas y desarrollo laboral.

**Resultados:**

En 26 países, una o más características de la atención primaria tenían potenciales de mejora medios o altos. El carácter integral de la atención tenía un potencial de mejora entre medio y alto en 23 de 34 países. En todos los países, la comunicación entre médico y paciente tenía un potencial de mejora bajo. Una estructura global más fuerte de la atención primaria se correlacionó con un menor potencial de mejora en la continuidad y exhaustividad de la atención. En los países con una política de dirección de la atención primaria más sólida, los pacientes percibieron un potencial menor de mejora de la continuidad de la atención. Los países con mejores condiciones económicas para la atención primaria presentaron un potencial menor para la mejora de todas las características de la atención centradas en la persona.

**Conclusión:**

En países con una estructura de atención primaria más sólida, los pacientes perciben un menor potencial de mejora de la atención primaria.

## ملخص

**الغرض:**

تحري تصورات المرضى حول احتمالات التحسين في مجال الرعاية الأولية في 34 بلداً.

**الطريقة:**

أجرينا مسحاً متعدد القطاعات على 69201 مريضاً قاموا للتو بزيارة الممارسين العموميين في مرافق الرعاية الأولية. وقام المرضى بتقييم خمس سمات للرعاية الأولية الشخصية - التوافر/الإتاحة والاستمرارية والشمولية وإشراك المرضى والتواصل بين الأطباء والمرضى. وقام عُشر المرضى بترتيب أهمية كل سمة من السمات باستخدام مقياس من واحد إلى أربعة وسجل تسعة أعشار المرضى خبراتهم بشأن تلقي الرعاية. وقمنا بحساب احتمالات التحسين بضرب نسبة الخبرات السلبية للمرضى بمتوسط درجة الأهمية في كل بلد. وتم تقسيم الدرجات إلى احتمالات تحسين منخفضة ومتوسطة ومرتفعة. وتم إيجاد الارتباطات الثنائية بين درجات التحسين والأبعاد الثلاثة لهيكل الرعاية الأولية وهي - تصريف الشؤون والظروف الاقتصادية وتنمية القوى العاملة.

**النتائج:**

حظيت واحدة أو أكثر من سمات الرعاية الأولية في 26 بلداً باحتمالات تحسين متوسطة أو مرتفعة. وحظيت شمولية الرعاية باحتمالات تحسين من متوسطة إلى مرتفعة في 23 بلداً من أصل 34 بلداً. وفي جميع البلدان، حظي التواصل بين الأطباء والمرضى باحتمالات تحسين منخفضة. وارتبط ازدياد هيكل الرعاية الأولية الأقوى بشكل عام بانخفاض احتمالات التحسين في الاستمرارية وشمولية الرعاية. وكان تصور المرضى في البلدان التي تتميز بتصريف شؤون أقوى فيما يتعلق بالرعاية الأولية هو انخفاض احتمالات التحسين في استمرارية الرعاية. وانخفضت احتمالات التحسين في جميع سمات الرعاية الشخصية لدى البلدان ذات الظروف الاقتصادية الأفضل للرعاية الأولية.

**الاستنتاج:**

في البلدان ذات هيكل الرعاية الأولية الأقوى، تتسم الرعاية الأولية باحتمالات تحسين أقل وفق تصورات المرضى.

## 摘要

**目的:**

调查34个国家病人对初级保健改善可能性的看法。

**方法:**

我们对最近前往初级保健设施的全科医生就医的69201名患者进行横断面调查。病人以个人为中心的初级保健的五个特性评级：可达性/可用性、连续性、综合性、病人参与和医患沟通。十分之一的患者按每个特性从一到四的重要性等级排名，十分之九的患者按所接受护理的体验评分。我们这样计算改善的可能性：负面病人体验比例乘以每个国家重要性平均值。改善可能性分数分为低、中、高等。在改善分数和初级保健结构的三个维度（治理、经济条件和人力发展）之间进行两两相关分析。

**结果:**

在26个国家，初级保健的一个或多个特性有中或高的改善可能性。在34个国家中有23个国家的护理综合性有中到高等的改善可能性。在所有国家中，医患沟通改善可能性较低。初级保健整体更强的结构与较低的护理连续性和综合性改善可能性相关。在初级保健治理更强的国家，患者认为不太可能改善医疗服务的连续性。初级护理经济条件更好的国家提高所有特性个人为中心护理的可能性更低。

**结论:**

在初级保健结构更强的国家，病人能意识到的初级保健改善可能性更低。

## Резюме

**Цель:**

Исследовать восприятие пациентами потенциала улучшения первичной медицинской помощи в 34 странах.

**Методы:**

Было проведено перекрестное исследование 69  201 пациента, которые посещали только терапевтов в учреждениях первичной медицинской помощи. Пациенты дали оценку пяти характеристикам целенаправленной первичной медицинской помощи: доступность/наличие, непрерывность, комплексность, участие пациента и коммуникация между врачом и пациентом. Одна десятая пациентов расположила по важности каждую характеристику на шкале от одного до четырех, а девять десятых пациентов оценили свой опыт получения медицинской помощи. Потенциал улучшения рассчитывался путем умножения части пациентов с отрицательным опытом на средний балл важности в каждой стране. Баллы делились на низкий, средний и высокий потенциал улучшения. Попарные корреляции выводились между баллами улучшения и тремя характеристиками структуры первичной медицинской помощи: руководством, экономическим положением и подготовкой трудовых ресурсов.

**Результаты:**

В 26 странах одна или более характеристик первичной медицинской помощи обладали средним или высоким потенциалом улучшения. Комплексность медицинской помощи обладала потенциалом улучшения от среднего до высокого в 23 из 34 стран. Во всех странах коммуникация между врачом и пациентом имела низкий потенциал улучшения. В целом сильная структура первичной медицинской помощи была связана с низким потенциалом улучшения непрерывности и комплексности медицинской помощи. В странах с эффективным руководством первичной медицинской помощью пациенты усматривали меньший потенциал для улучшения непрерывности медицинской помощи. Страны с лучшим экономическим положением в первичной медицинской помощи обладали меньшим потенциалом улучшения всех характеристик целенаправленной помощи пациенту.

**Вывод:**

В странах с эффективной структурой первичной медицинской помощи пациенты усматривали меньший потенциал улучшения в данной области.

## Introduction

Due to the increased prevalence of comorbid conditions, people often have more than one disease that needs to be managed consistently over time.^1^^,^^2^ Health-care providers can do this through a person-focused approach, which entails goal-oriented, rather than disease-oriented care. The goal is to manage people’s illnesses through the course of their life.^1^^,^^2^ Therefore, person-focused care should be continuous, accessible and comprehensive. It should also be coordinated when patients have more than one provider.^1^

Patients’ assessment of health care can be divided into what patients find important and what they have experienced.^3^^–^^5^ Importance refers to what people see as desired features of health care – i.e. patients’ instrumental values.^6^ The combination of instrumental values and patients’ experiences constitute quality judgments, which provides insight on the extent to which health-care providers meet these values. Both instrumental values and experiences of primary care patients vary between countries.^6^^–^^8^ These judgements can be transformed into a measure of improvement potential. When an aspect of care is experienced as poorly performed, but not considered important, this can be seen as less of a quality problem than if patients consider the aspect important.^9^ More important aspects of care thus have higher improvement potential.

The structure of primary care can relate to person-focused care in various ways. In stronger primary care structures the providers are more likely to be involved in a wide range of health problems at different stages of the patients’ lives. This is expected to increase continuity of care and providers’ responsiveness to the patients’ values regarding continuity, comprehensiveness and communication. Patients will use services more readily if they know a broad spectrum of care is offered.^10^ A stronger primary care structure is associated with more accessible primary care,^11^ which is one of the core features of person-focused care. Therefore, we expect that in countries with a stronger primary care structure, the patient-perceived improvement potential of person-focused primary care is lower.

The primary care structure comprises governance, economic conditions such as the mode of financing of providers and expenditures on primary care, and workforce development – the profile and the education of the primary-care providers.^12^^,^^13^

**Fig. 1:**
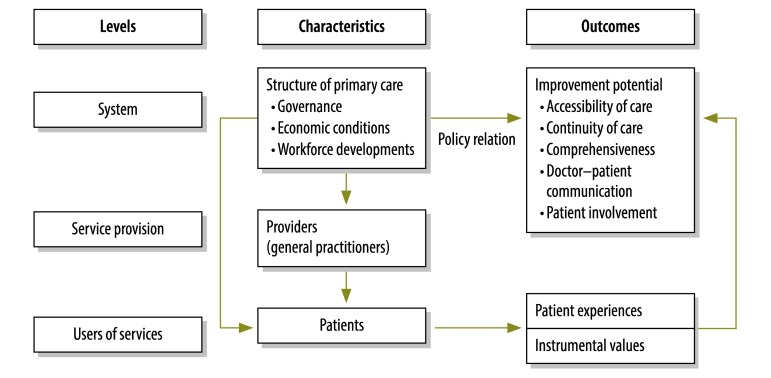
Features that influence the extent to which primary care is person-focused

Note: Instrumental value is what the patient finds important.

We wished to quantify the extent to which the structure of primary care at the national level in 34 countries is related to patient-perceived improvement potential for features of person-focused care. To study this relationship, the empirical relations between the providers – general practitioners – and patients need to be considered (Fig. 1). The primary care structure influences the behaviour of the practitioners, which will influence patients’ experiences. Patients’ characteristics – e.g. age and income – influence patients’ individual experiences and values. We focus on the system level to study characteristics that are amenable to policy interventions.

## Methods

We derived aggregated data on patient-perceived improvement potential in 34 countries from the QUALICOPC study (Quality and Costs of Primary Care in Europe). In this study, patients in 31 European countries (Austria, Belgium, Bulgaria, Cyprus, Czech Republic, Denmark, Estonia, Finland, Germany, Greece, Hungary, Iceland, Ireland, Italy, Latvia, Lithuania, Luxembourg, Malta, the Netherlands, Norway, Poland, Portugal, Romania, Slovakia, Slovenia, Spain, Sweden, Switzerland, the former Yugoslav Republic of Macedonia, Turkey, the United Kingdom of Great Britain and Northern Ireland) responded to surveys. Three non-European countries (Australia, Canada, New Zealand) were also included. In each country, patients of general practitioners filled in the questionnaires (target: *n* = 2200 per country; Cyprus, Iceland and Luxembourg *n* = 800). In Belgium, Canada, Spain and Turkey, larger samples were taken to enable comparisons between regions (Table 1). We aimed to get a nationally representative sample of general practitioners. If national registers of practitioners were available, we used random sampling to select practitioners. In countries with only regional registers, random samples were drawn from regions that represented the national setting. If no registers existed, but only lists of facilities in a country, a random selection from such lists was made. The patients of only one practitioner per practice or health centre were eligible to participate. Details of the study protocol have been published elsewhere.^14^^,^^15^

**Table 1:** Overview of the survey investigating the potential for improvement of primary care in 34 countries, 2011–2013

Country	No. of general practitioners facilities^a^	No. of patient experience questionnaires completed	No. of patient values’ questionnaires completed	Relative strength of primary care structure^b^
Australia	133	1190	138	Strong
Austria	180	1596	188	Medium
Belgium	411	3677	407	Medium
Bulgaria	221	1991	222	Weak
Canada	553	5009	806	Strong
Cyprus	71	624	71	Weak
Czech Republic	220	1980	220	Weak
Denmark	212	1878	209	Strong
Estonia	128	1121	126	Medium
Finland	139	1196	129	Medium
Germany	237	2117	234	Medium
Greece	221	1964	219	Weak
Hungary	221	1934	215	Weak
Iceland	90	761	82	Weak
Ireland	191	1694	186	Medium
Italy	219	1959	220	Strong
Latvia	218	1951	212	Medium
Lithuania	225	2011	224	Medium
Luxembourg	80	713	79	Weak
Malta	70	626	68	Weak
Netherlands	228	2012	222	Strong
New Zealand	131	1150	197	Strong
Norway	203	1529	175	Medium
Poland	220	1975	219	Weak
Portugal	212	1920	215	Strong
Romania	220	1975	220	Strong
Slovakia	220	1918	220	Weak
Slovenia	219	1963	216	Strong
Spain	433	3731	431	Strong
Sweden	88	773	112	Medium
Switzerland	200	1791	198	Weak
The former Yugoslav Republic of Macedonia	143	1283	143	Medium
Turkey	290	2623	292	Medium
United Kingdom^c^	160	1296	155	Strong

^a^ Patients of one general practitioner per facility were surveyed.

^b^ Based on Kringos et al. 2013.^11^

^c^ Only patients in England were surveyed.

In nearly all countries (30), trained fieldworkers were sent to the participating practices to collect patient data using paper questionnaires. In Canada, Denmark, New Zealand, the United Kingdom and parts of Norway and Sweden, the practice staff were instructed to distribute and collect the questionnaires. The fieldworkers and practice staff were instructed to invite consecutive patients, who had had a face-to-face consultation with the practitioner and who were 18 years or older, to complete the questionnaire until 10 questionnaires per practice were collected. Of these 10 questionnaires, nine assessed the experiences in the consultation which had just occurred and one questionnaire included questions about the patient’s primary care values. The proportions of the questionnaires were based on the findings that, within a country, patients’ experiences varied widely but there was little variation in what the patients found important.^7^ In the patient experience questionnaire, patients were asked to indicate whether they agreed with a statement by selecting “Yes” or “No” answers. For example, the proportion of negative experiences for the statement “during the consultation the doctor had my medical records at hand” would be the proportion stating that the doctor did not have the medical records at hand. In the patient values’ questionnaire – which contained the same questions as the patient experience questionnaire – patients could indicate the importance of a statement, e.g. the importance of the doctor having medical records at hand, by selecting “not important”, “somewhat important”, “important” or “very important”. The answers were scored, ranging from 1 (not important) to 4 (very important). Missing answers were excluded from the calculations.

Ethical approval was acquired in accordance with the legal requirements in each country. The surveys were carried out anonymously. Data collection took place between October 2011 and December 2013. The patient experience questionnaire was filled in by 61 931 patients and the patient values’ questionnaire by 7270 patients. Appendices A and B contain the questionnaires (available at: http://www.nivel.nl/pdf/Appendices-Assesing-the-potential-for-improvement-of-PC-in-34-countries-WHO-Bulletin-2015.pdf).

### Operationalization of concepts

#### Dependent variables

As an outcome indicator for health care, we used the patient-perceived improvement potential, which is based on the consumer quality (CQ) index, a validated and standardized measurement instrument.^16^ Person-focused primary care was measured using 16 items, such as whether the practitioner displayed knowledge about the patient’s personal living circumstances. The items were derived from the CQ index for general practice and tested in the QUALICOPC pilot study.^15^^,^^17^ Improvement potential was expressed in improvement scores, which are calculated by multiplying the proportion of negative experiences for each question – the answers which indicate lower quality – with the value scores of the corresponding statement per country. The value score was calculated by taking the mean value for each country on a scale from one to four. A higher improvement score indicates a higher need for improvement.

The improvement potential of each country was measured for the following main features: accessibility/availability (five questions), continuity (three questions), comprehensiveness (two questions), patient involvement (one question) and doctor–patient communication (five questions). For each feature, a mean patient-perceived improvement score was calculated. Based on the range of scores found (0.11–1.95) the level of improvement potential is considered relatively low (0.11–0.72), medium (0.73–1.34) or high (1.35–1.95).

#### Independent variables

For 30 countries (Australia, Canada, New Zealand and the former Yugoslav Republic of Macedonia were excluded), we collected data from the Primary Health Care Activity Monitor (PHAMEU) study on a set of indicators for the dimensions of governance, economic conditions and workforce development of the primary care structure.^18^ Examples of such indicators are the availability of evidence-based guidelines for general practitioners (governance) and the percentage of medical universities with a postgraduate programme in family medicine (workforce development).^18^ The PHAMEU database provides scores indicating the strength of each indicator, ranging from 1 (weak) to 3 (strong) and overall scale scores for each dimension, calculated using a two-level hierarchical latent regression model, and an overall structure score combining the three dimensions.^11^ Additionally, we collected data for Australia, Canada, New Zealand and the former Yugoslav Republic of Macedonia using the same methods as for the PHAMEU study. Table 1 lists the relative strength of each countries’ primary care structure, Appendix C contains the indicators and Appendix D contains scale scores per dimension.

### Statistical analyses

One-tailed pairwise correlations were used to measure the associations between the independent and dependent variables, because the hypothesis has one direction, namely that a stronger primary care structure is associated with more person-focused care. *P* < 0.05 was considered statistically significant.

Sensitivity analyses were done using an alternative method of analysis for the improvement scores. Multilevel analyses were used to calculate country-level scores of the experience and values items, using the country level residuals of the items. The scores were adjusted for several variables at the practitioner and patient level (e.g. age and gender of the general practitioners and patients). When comparing the raw improvement scores and the ones calculated on the basis of multilevel residuals no significant differences were found. Correlation coefficients between the raw improvement scores as used in this paper and the adjusted improvement scores were above 0.91.

In the PHAMEU conceptual model and corresponding database, gatekeeping (practitioners determining the necessity for referral of patients to other levels of the health system) is considered to be part of the process of primary care. However, in previous studies, gatekeeping has been used as a potential determinant of primary care performance. Therefore, additional sensitivity analysis was performed on the association between the improvement potential and gatekeeping. The results of this analysis are presented in Appendix E. Analyses were carried out using Stata version 13.0 (StataCorp. LP, College Station, United States of America) and MLWin version 2.25 (University of Bristol, Bristol, United Kingdom).

## Results

### Improvement potential

In total, 69 201 patients completed the questionnaire and the average response rate was 74.1% (range: 54.5%–87.6%). A detailed overview of the patients’ experience scores, values’ scores and patient-perceived improvement scores per country are provided in Appendices F–H. The background characteristics of the patients can be found in Appendix I.

For accessibility of care, five countries – Cyprus, Portugal, Slovakia, Spain and Turkey – showed a medium level of improvement potential. The remaining countries showed a low improvement potential. While most of the countries were found to have a low improvement potential regarding the continuity of care, Greece, Malta and Turkey show a medium level and Cyprus a high level. Comprehensiveness of care showed a medium level of patient-perceived improvement potential in 20 countries and a relatively high level in Cyprus, Malta and Sweden. Patients’ involvement in decision-making about their treatment had a medium level of improvement potential in nine countries and a high level in Cyprus. In all countries, values were relatively low for doctor–patient communication, indicating that the primary-care providers meet their patients’ expectations in this domain (Table 2).

**Table 2:** Mean patient-perceived improvement scores for primary care in 34 countries, 2011–2013

Country	Improvement score^a^				
	Accessibility	Continuity	Comprehensiveness	Involvement	Communication
Australia	0.38	0.14	0.42	0.17	0.16
Austria	0.41	0.38	0.97	0.65	0.20
Belgium	0.34	0.26	0.57	0.26	0.22
Bulgaria	0.66	0.56	1.34	1.17	0.34
Canada	0.38	0.11	0.52	0.18	0.12
Cyprus	1.25	1.40	1.95	1.47	0.38
Czech Republic	0.44	0.26	1.00	0.79	0.18
Denmark	0.26	0.18	0.82	0.56	0.23
Estonia	0.40	0.22	0.87	0.80	0.22
Finland	0.46	0.36	0.81	0.55	0.21
Germany	0.33	0.27	0.81	0.50	0.20
Greece	0.72	1.08	0.70	0.77	0.24
Hungary	0.49	0.49	1.05	0.48	0.30
Iceland	0.53	0.24	1.14	0.46	0.24
Ireland	0.45	0.26	0.72	0.66	0.37
Italy	0.51	0.31	0.91	0.76	0.42
Latvia	0.51	0.26	0.67	0.70	0.40
Lithuania	0.52	0.38	0.62	0.84	0.24
Luxembourg	0.39	0.31	0.62	0.57	0.23
Malta	0.60	1.17	1.36	0.65	0.33
Netherlands	0.30	0.25	0.91	0.47	0.28
New Zealand	0.22	0.11	0.52	0.18	0.12
Norway	0.52	0.31	0.93	0.52	0.21
Poland	0.55	0.56	1.02	0.90	0.23
Portugal	0.73	0.19	0.50	0.73	0.27
Romania	0.55	0.30	1.04	0.65	0.29
Slovakia	0.74	0.53	1.12	0.63	0.28
Slovenia	0.53	0.32	1.16	0.78	0.23
Spain	0.90	0.29	1.16	0.57	0.36
Sweden	0.54	0.62	1.38	0.60	0.27
Switzerland	0.27	0.18	0.60	0.27	0.16
The former Yugoslav Republic of Macedonia	0.38	0.23	0.92	0.61	0.14
Turkey	0.77	0.84	1.06	0.38	0.36
United Kingdom^b^	0.42	0.30	0.77	0.47	0.21

^a^ The improvement score was calculated by multiplying the proportion of negative patient experiences with the mean importance score.

^b^ Only patients in England were surveyed.

Note: Scores between 0.11–0.72 were considered as a low level of patient-perceived improvement potential. Scores between 0.73–1.34 were considered as a medium level of patient-perceived improvement potential. Scores between 1.35–1.95 were considered as a high level of patient-perceived improvement potential.

The relatively high levels of patient-perceived improvement potential in Cyprus – three features with high potential and one feature with medium – indicate weak performance of primary care. In Turkey, three areas showed a medium level of patient-perceived improvement potential. Countries showing relatively low improvement potential in all features were Australia, Belgium, Canada, Ireland, Latvia, Luxembourg, New Zealand and Switzerland, indicating that primary care in these countries is perceived as person-focused.

### Primary care structure

The patient-perceived improvement potential for continuity and comprehensiveness of care had a significant negative association with the overall structure of primary care. If a country has a stronger primary care structure, primary care is more person-focused for these features. For the separate structural dimensions, patients’ perceived care to be more continuous in countries with stronger primary care governance. Stronger economic conditions in primary care were found to be associated with all features of person-focused care. Although workforce development correlated negatively with all features, none of the values were significantly correlated (Table 3).

**Table 3:** Correlations between the strength of primary care structure and patient perceived improvement scores in 34 countries, 2011–2013

Feature	Primary care structure			
	Overall	Governance	Economic conditions	Workforce development
Accessibility	−0.2562	−0.1136	−0.3187*	−0.2244
Continuity	−0.3962*	−0.3320*	−0.3833*	−0.2263
Comprehensiveness	−0.3230*	−0.1739	−0.3663*	−0.269
Involvement	−0.2833	−0.0484	−0.5768*	−0.2772
Communication	−0.1202	−0.0475	−0.3720*	−0.0513

*P < 0.05 (one-tailed).

In eight countries where patient-perceived improvement potential is relatively low, the overall strength of the primary care structure varies. The relative strength is strong in Australia, Canada and New Zealand, medium in Belgium, Ireland and Latvia and weak in Luxembourg and Switzerland. The strongest associations between strength and improvement potential were found for economic conditions for primary care. These conditions are relatively strong in Australia, Belgium and New Zealand and medium in Latvia and Switzerland.

## Discussion

This study evaluates the extent to which primary care in 34 countries is person-focused by asking patients of general practitioners about what they find important and their actual experiences. The combination of these aspects provides us with insight on what patients perceive as priority improvement areas. In most countries primary care shows one or more features with a medium or high level of patient-perceived improvement potential. Accessibility and continuity of care show relatively low improvement potential, while in many countries comprehensiveness is indicated as a priority area. In this study, comprehensiveness of care indicates whether general practitioners ask their patients about additional problems and whether there is opportunity to discuss psychosocial problems. Our results confirm previous studies showing that practitioners perform well on general aspects of communication.^19^^–^^21^ One explanation for this result could be the on-going relationship between practitioners and their patients. Larger variations have been found between countries on the relevance of communication and practitioners’ performance for specific issues.^22^ Eight countries showed low improvement potential in all features, indicating positive patient experiences. Previous studies in Australia and New Zealand have also found positive patient experiences.^23^^,^^24^ Another study comparing 10 European countries, found positive patient assessments in Belgium, Germany and Switzerland and less positive assessments in the United Kingdom and the Scandinavian countries.^21^ This is largely in line with our findings.

We could largely confirm the hypothesis that a stronger primary care structure is associated with more person-focused care. Stronger structures were associated with more continuous and comprehensive care. Continuity is an important aspect of person-focused care. Stronger governance is also associated with more continuity. In countries with stronger economic conditions for primary care we found less improvement potential in all areas.

The sensitivity analysis for the association between gatekeeping and patient-perceived improvement potential showed that gatekeeping was associated only with lower perceived improvement potential for continuity of care.

Strengths of this study were the inclusion of data from many countries and that patients were asked about their actual experiences immediately after the consultation with their practitioners. There were also limitations. First, there are countries where other providers offer primary care besides general practitioners. These providers were not included in this study. Second, only the actual visitors to general practices were surveyed. This means that we do not have information about the people who do not have access to such practices. In all countries, improvement potential for accessibility of care might be higher than measured in this study. For example, a report based on the Canadian QUALICOPC data found that patient-reported access in this study is more positive compared to other previous studies.^25^^–^^28^ Third, in Greece, most participating general practitioners worked in health centres, while there are also many practitioners in Greece working outside health centres. Comparing different countries should be done cautiously, since the extent to which general practitioners are involved in primary care and the types of illnesses they treat differs between countries.

When measuring instrumental values and experiences of patients, people may judge importance by what they have already experienced in health care.^6^ For example, when practitioners in a country perform poorly on a certain aspect, patients might have lower expectations and will find this aspect less important. Experiences and values of patients have been found to be correlated,^6^ perhaps because patients seek health-care providers who deliver care according to their values.

The World Health Organization advocates for primary care that puts people first. A stronger primary care structure is necessary to make progress towards this goal.^10^
